# Coding and noncoding transcriptomes of NODULIN HOMEOBOX (NDX)-deficient *Arabidopsis* inflorescence

**DOI:** 10.1038/s41597-023-02279-9

**Published:** 2023-06-07

**Authors:** Orsolya Feró, Zsolt Karányi, Éva Nagy, Ágnes Mosolygó-L, Henrik Mihály Szaker, Tibor Csorba, Lóránt Székvölgyi

**Affiliations:** 1 MTA-DE Momentum, Genome Architecture and Recombination Research Group, Faculty of Pharmacy, University of Debrecen, Debrecen, H-4032 Hungary; 2 Department of Internal Medicine, Faculty of Medicine, University of Debrecen, Debrecen, H-4032 Hungary; 3 Department of Biochemistry and Molecular Biology, Faculty of Medicine, University of Debrecen, Debrecen, H-4032 Hungary; 4 MATE University, Genetics and Biotechnology Institute, Gödöllő Pest, H-2100 Hungary; 5 Institute of Plant Biology, Biological Research Centre, Szeged, H-6726 Hungary

**Keywords:** Gene expression, Plant molecular biology

## Abstract

*Arabidopsis* NODULIN HOMEOBOX (NDX) is a plant-specific transcriptional regulator whose role in small RNA biogenesis and heterochromatin homeostasis has recently been described. Here we extend our previous transcriptomic analysis to the flowering stage of development. We performed mRNA-seq and small RNA-seq measurements on inflorescence samples of wild-type and *ndx1-4* mutant (WiscDsLox344A04) *Arabidopsis* plants. We identified specific groups of differentially expressed genes and noncoding heterochromatic siRNA (hetsiRNA) loci/regions whose transcriptional activity was significantly changed in the absence of NDX. In addition, data obtained from inflorescence were compared with seedling transcriptomics data, which revealed development-specific changes in gene expression profiles. Overall, we provide a comprehensive data source on the coding and noncoding transcriptomes of NDX-deficient *Arabidopsis* flowers to serve as a basis for further research on NDX function.

## Background & Summary

NODULIN HOMEOBOX (NDX) is a specific member of the homeobox family of transcription factors in flowering plants^1,2^, particularly in the *Brassicaceae* family, which includes the genetic model *Arabidopsis thaliana*. Besides its homeobox domain, NDX has two atypical domains called NDX-A and NDX-B, whose molecular functions are less understood. Recent molecular data indicate that NDX regulates the activity of some euchromatic genes in *Arabidopsis thaliana* plants. NDX was shown to interact with the E3 ubiquitin ligase module of the Polycomb Repressive Complex 1 (PRC1), RING1A and RING1B, establishing a functional link between histone H2A ubiquitination (H2Aub) and abscisic acid (ABA)-mediated repressive chromatin signalling^3^. Through the ABA pathway, NDX regulates seed germination and root growth. NDX was also shown to interact with the transcriptional repressor VIVIPAROUS1/ABI3-LIKE (VAL1) (through binding RING1A and RING1B), which regulates the activity of FLOWERING LOCUS C (FLC), a central integrator of the flowering transition in *Arabidopsis*^4^.

In addition to the above functions, NDX exhibits significant binding affinity to different nucleic acid substrates^3,5^. NDX was shown to bind to an R-loop structure at the 3′-end of FLC, which serves as a promoter for antisense (*COOLAIR*) transcription. *COOLAIR* is a set of antisense long noncoding RNAs that act as a repressor of FLC. The association of NDX and *COOLAIR* suggested a model in which *COOLAIR* transcription was repressed by NDX-mediated R-loop stabilization, which in turn altered FLC expression and flowering time.

Recent genomic data challenged the idea that NDX acts as a general R-loop regulator that controls the formation of chromosomal R-loops throughout the *Arabidopsis* genome. Instead, NDX appears to be linked to heterochromatin function by regulating the activity of heterochromatic siRNAs (het-siRNAs) and non-CG DNA methylation pathways at pericentromeric regions^6^. This observation is consistent with earlier data showing that NDX coincides with a heterochromatic patch of H3K9 dimethylation and Pol IV-dependent siRNA transcripts within the FLC terminator/*COOLAIR* promoter region^5,7^, linking NDX to heterochromatin function.

**Fig. 1:**
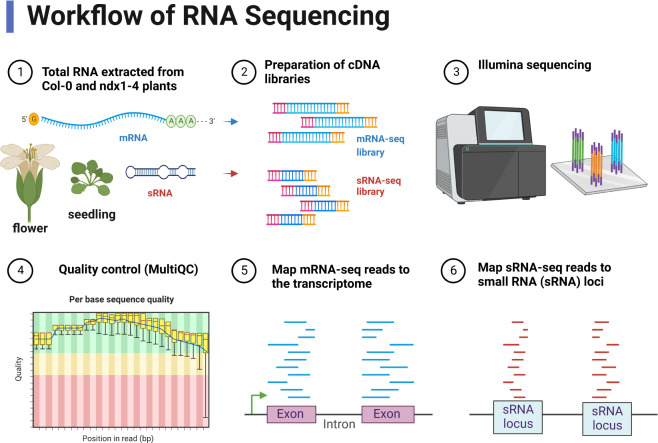
General workflow of mRNA-seq and sRNA-seq experiments performed in *Arabidopsis thaliana*. Two different tissue types were analysed: flowers and seedlings. The seedling data were published in ref. ^6^, which were used for comparison with the flower transcriptomic data generated in this study.

The molecular phenotype supporting the role of NDX in heterochromatin homeostasis was identified in genomic data of 10-day-old seedlings, however, similar functional studies were not performed in other stages of plant development. In particular, there is a lack of transcriptome data in *Arabidopsis* inflorescence, which awaits to be generated to understand the effect of NDX on the expression of flowering-related coding and noncoding RNAs. Herein, we extend our transcriptomic measurements to the flowering stage and present novel mRNA-seq and small RNA (sRNA)-seq data on inflorescence tissues of wild-type and *ndx1-4* T-DNA insertion mutant (WiscDsLox344A04) plants (Fig. 1). Our analysis identified specific sets of genes differentially expressed in *ndx1-4*, as well as noncoding heterochromatic siRNA (het-siRNA) loci whose activity was significantly changed in the absence of NDX. Transcriptomic data obtained from inflorescence were also compared with previously published seedling data^6^, highlighting development-specific changes in gene expression profiles.

## Methods

*Arabidopsis thaliana* Col-0 wild-type accession and *ndx1-4* mutant (WiscDsLox344A04)^5^ plants were directly sown on soil and grown side-by-side at 21 °C on long day conditions (LD, 16 h light, 8 h dark photoperiod) until flowering stage. After five weeks, when the central flowering stem of plants reached approximately 20–25 cm and side branches were also present, inflorescence tissues were collected. Total RNA isolation was performed by the standard phenol-chloroform extraction method as described previously^8^. Briefly, approximately 30 mg plant material per sample was homogenized and resuspended in 600 μl of extraction buffer (0.1 M glycine-NaOH, pH 9.0, 100 mM NaCl, 10 mM EDTA, 2% SDS). The sample was mixed with an equal volume of phenol (pH 4.3, Sigma-Aldrich, P3803). The aqueous phase was treated with 600 μl of phenol-chloroform and chloroform, precipitated with ethanol. RNA samples were eluted in nuclease-free water and used in subsequent steps. To remove genomic contaminations, Dnase I treatments were performed based on manufacturer’s instructions (Ambion AM2222, www.thermofisher.com); RNA quality was assessed by testing RNA degradation based on agarose gel electrophoresis and presence of contaminants by Nanodrop spectrophotometer measurements (260/230 and 260/280 ratios). RNA quality was further assayed by capillary electrophoresis (Agilent 2100).

**Fig. 2:**
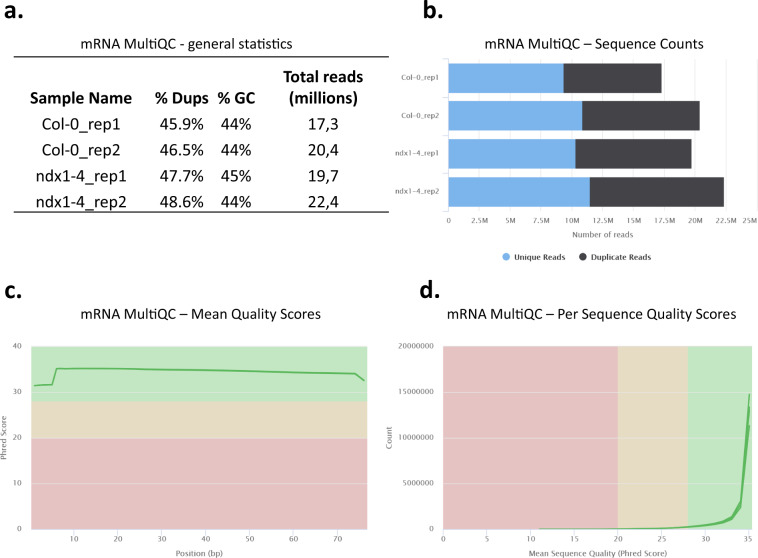
MultiQC analysis of mRNA-seq data from flowers. (**a**) General NGS statistics of mRNA-seq samples. (**b**) Sequence Counts plot showing the total number of reads, classified as unique and duplicate. Duplication detection requires an exact sequence match over the whole length of the sequence. Any reads over 75 bp in length were truncated to 50 bp for this analysis. More about duplicate calculation: https://multiqc.info/. (**c**) Mean quality scores. The higher the score, the better the base call. Background color of the graph divides the y axis into very good quality calls (green zone), calls of reasonable quality (orange zone), and calls of poor quality (red zone). (**d**) Per sequence quality scores. The graph shows if a subset of sequences have universally low-quality values. Background color: of the graph very good quality calls (green), calls of reasonable quality (orange), and calls of poor quality (red).

For RNA-seq, cDNA libraries were prepared from two independent biological replicates according to Illumina’s TruSeq RNA Library Preparation Kit v2 protocol. Equal amounts of total RNA extracts from 5 inflorescence tissues of different plants were pooled (to create one pool) for each biological replicate (see Tables 1, 2 for description of samples). The mRNA-seq libraries were sequenced using an Illumina NextSeq 500 instrument with 1 × 75 bp reads, generating 17.3–22.4 million reads per sample (Fig. 2a). Raw sequence data quality was assessed using FastQC and summarized reports were generated with MultiQC^9^. Duplication detection indicated >50% of reads as unique (Fig. 2b). Mean Phred quality scores for each read position in each mRNA sample were higher than 28 (indicative of ‘very good quality’) (Fig. 2c). Per sequence mean Phred quality scores were higher than 28 (indicative of ‘very good quality’) for at least 95% of reads in each mRNA sample (Fig. 2d).

**Table 1:** List of NGS experiments, result tables and identifiers of inflorescence samples used in the current study.

Data description	Tissue	Sample description	SRA/GEO reference	Data collection/Analytical step/Figure
mRNA-seq (fastq)	flower	Col-0 wt, rep1	SRX19147910	mRNA sequencing, 75 bp single-end reads; Fig. 2
		Col-0 wt, rep2	SRX19147911	
		ndx1-4 mt, rep1	SRX19147912	
		ndx1-4 mt, rep2	SRX19147913	
mRNA coverage (bigwig)	flower	Col-0 wt, rep1	GSM6965098	HISAT2: alignment to TAIR10 reference; deepTools: RPKM normalized read coverage; JBrowse
		Col-0 wt, rep2	GSM6965099	
		ndx1-4 mt, rep1	GSM6965100	
		ndx1-4 mt, rep2	GSM6965101	
differentially expressed genes (tsv)	flower	ndx1-4 vs Col-0	GSE223589	Salmon: quantification with GC bias correction; DESeq2: differential gene expression analysis, adjusted p-value < 0.01; data: counts, FPKM, log2FC, p-value (adjusted); Fig. 3
differentially expressed transposable elements (tsv)	flower	ndx1-4 vs Col-0	GSE223589	Salmon: quantification with GC bias correction, keeping duplicates; DESeq2: differential transposable element expres- sion analysis, adjusted p-value < 0.05; data: counts, FPKM, log2FC, p-value (adjusted)
sRNA-seq (fastq)	flower	Col-0 wt, rep1	SRX19147839	sRNA sequencing, 50 bp single-end reads; Figure 6
		Col-0 wt, rep2	SRX19147840	
		Col-0 wt, rep3	SRX19147841	
		ndx1-4 mt, rep1	SRX19147842	
		ndx1-4 mt, rep2	SRX19147843	
		ndx1-4 mt, rep3	SRX19147844	
sRNA coverage (bigwig)	flower	Col-0 wt, rep1	GSM6965102	bowtie2: alignment to TAIR10 reference; deepTools: RPKM normalized read coverage; JBrowse
		Col-0 wt, rep2	GSM6965103	
		Col-0 wt, rep3	GSM6965104	
		ndx1-4 mt, rep1	GSM6965105	
		ndx1-4 mt, rep2	GSM6965106	
		ndx1-4 mt, rep3	GSM6965107	
differentially expressed sRNAs (tsv)	flower	ndx1-4 vs Col-0	GSE223590	DESeq2: differential sRNA expression analysis, adjusted p-value < 0.05; data: counts, log2FC, p-value (adjusted); Fig. 7 (cutoff: abs(FC) > 1.5)

**Table 2:** List of NGS experiments, result tables and identifiers of seedling samples used in the current study.

Data description	Tissue	Sample description	SRA/GEO reference	Data collection/Analytical step/Figure
mRNA-seq (fastq)	seedling	Col-0 wt, rep1	SRX16109944	mRNA sequencing, 50 bp single-end reads
		Col-0 wt, rep2	SRX16109945	
		ndx1-4 mt, rep1	SRX16109946	
		ndx1-4 mt, rep2	SRX16109947	
mRNA coverage (bigwig)	seedling	Col-0 wt, rep1	GSM6320929	HISAT2: alignment to TAIR10 reference; deepTools: RPKM normalized read coverage
		Col-0 wt, rep2	GSM6320930	
		ndx1-4 mt, rep1	GSM6320931	
		ndx1-4 mt, rep2	GSM6320932	
differentially expressed genes (tsv)	seedling	ndx1-4 vs Col-0	GSE207842	Salmon: quantification with GC bias correction; DESeq2: differential gene expression analysis, adjusted p-value < 0.01; data: counts, FPKM, log2FC, p-value (adjusted)
differentially expressed genes (tsv)	flower/seedling	Col-0 flower vs Col-0 seedling	GSE223589	Salmon: quantification with GC bias correction; DESeq2: differential gene expression analysis, adjusted p-value < 0.01; data: counts, FPKM, log2FC, p-value (adjusted); Fig. 4
differentially expressed genes (tsv)	flower/seedling	ndx1-4 flower vs ndx1-4 seedling	GSE223589	Salmon: quantification with GC bias correction; DESeq2: differential gene expression analysis, adjusted p-value < 0.01; data: counts, FPKM, log2FC, p-value (adjusted); Fig. 5
differentially expressed transposable elements (tsv)	seedling	ndx1-4 vs Col-0	GSE207842	Salmon: quantification with GC bias correction, keeping duplicates; DESeq2: differential transposable element expres- sion analysis, adjusted p-value < 0.05; data: counts, FPKM, log2FC, p-value (adjusted)
sRNA-seq (fastq)	seedli g	Col-0 wt, rep1	SRX15045416	sRNA sequencing, 50 bp single-end reads
		Col-0 wt, rep2	SRX15045417	
		Col-0 wt, rep3	SRX15045418	
		ndx1-4 mt, rep1	SRX15045419	
		ndx1-4 mt, rep2	SRX15045420	
		ndx1-4 mt, rep3	SRX15045421	
differentially expressed sRNAs (tsv)	seedling	ndx1-4 vs Col-0	GSE201840	DESeq2: differential sRNA expression analysis, adjusted p-value < 0.05; data: counts, log2FC, p-value (adjusted)

Next, Salmon^10^ was used to map mRNA-seq reads and get transcript quantities for protein coding genes, noncoding genes and transposable elements. Reads were mapped to the *Arabidopsis thaliana* reference transcriptome (Araport11, coding and noncoding genes) to quantify gene expression levels as well as to transposable elements (Araport11) to quantify transposable element expression (see Table 3 for detailed description of *Arabidopsis thaliana* reference data used in the analyses). Transcript quantities of protein coding genes, noncoding genes, and transposable elements were corrected for potential fragment level GC bias using ‘salmon quant–gcBias’ that was shown to reduce isoform quantification errors^10,11^. Duplicate reads were filtered out from alignment to protein coding and noncoding genes (default behaviour of ‘salmon quant’) while duplicates were kept for transposable element alignments (‘salmon quant’ was used with ‘–keepDuplicates’ option set).

**Table 3:** List of *Arabidopsis thaliana* reference data used in this study.

Data description	ID/version
Reference sequence	TAIR10
Sequences of protein coding genes	Araport11, version 201606
Sequences of noncoding genes	Araport11, version 201606
Annotation (genes and transposons)	Araport11, version 201606
small RNA loci database	PRJEB22276
small RNA loci additional material	GitHub - seb-mueller/Arabidopsis_smallRNA_loci

DESeq2 was employed to identify differentially expressed genes (DEGs) and transposable elements (ndx1-4 versus Col-0 wild type)^12^. The significance level was established based on the adjusted p-values with independent hypothesis weighting^13^, where p(adjusted) <0.01 was used to define DEGs and p(adjusted) <0.05 for differentially expressed transposable elements. For the purpose of data visualization, RNA-seq reads were aligned to the TAIR10 reference genome using HISAT2^14^, which allowed for reporting spliced alignments. To create RPKM (Reads Per Kilobase per Million) normalized coverage files, we utilized deepTools bamCoverage^15^.

**Fig. 3:**
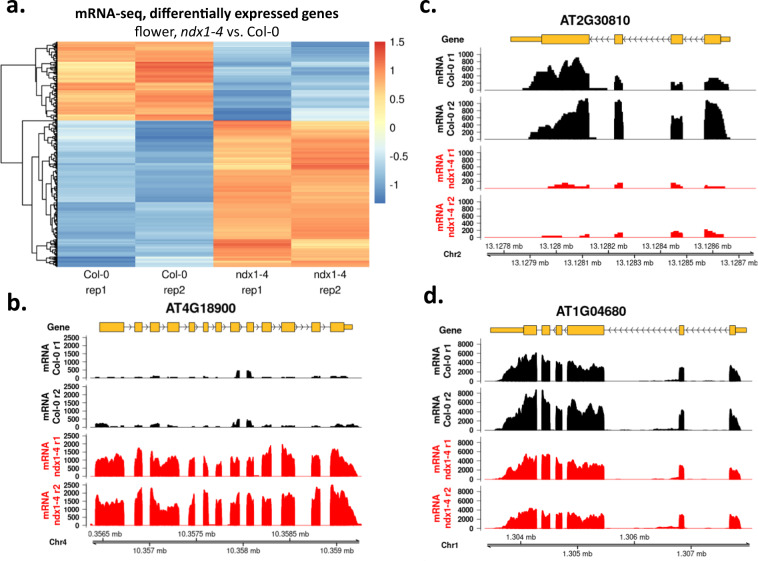
Differentially expressed genes in Col-0 and *ndx1-4* mutant *Arabidopsis* inflorescence. (**a**) Hierarchical clustering of log_2_ fold change values (*ndx1-4* vs. Col-0) calculated by DESeq2 analysis. Orange shows upregulated genes, blue shows downregulated genes. Two independent biological replicates are shown. (**b**) Representative screenshots of upregulated genes in *ndx1-4* flowers. (**c**) Representative screenshots of downregulated genes in *ndx1-4* flowers. (**d**) Representative screenshots of genes showing no differential expression in *ndx1-4* flowers.

**Fig. 4:**
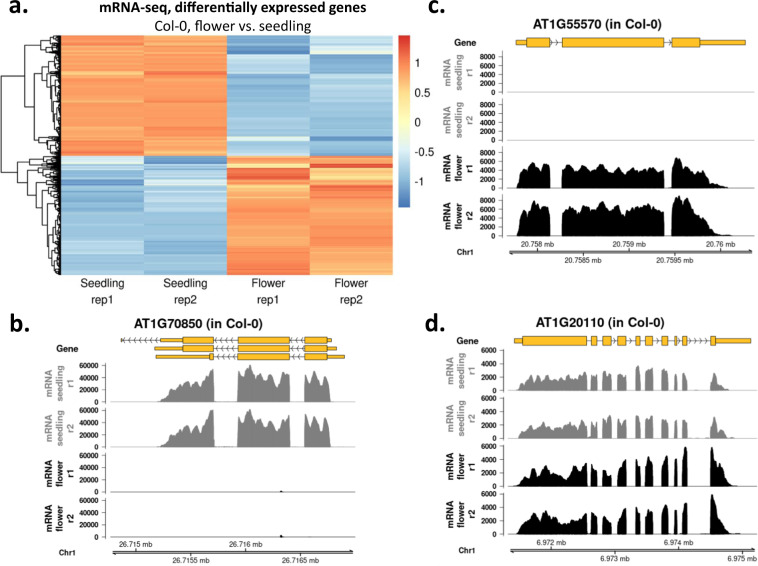
Differentially expressed genes in Col-0 seedlings and flowers. (**a**) Hierarchical clustering of log_2_ fold change values (seedling, Col-0 vs. flower, Col-0) calculated by DESeq2 analysis. Orange shows upregulated genes, blue shows downregulated genes. Two independent biological replicates are shown. (**b**) Representative screenshots of downregulated genes in flowers. (**c**) Representative screenshots of upregulated genes in flowers. (**d**) Representative gene showing no differential expression in Col-0 seedlings and flowers.

**Fig. 5:**
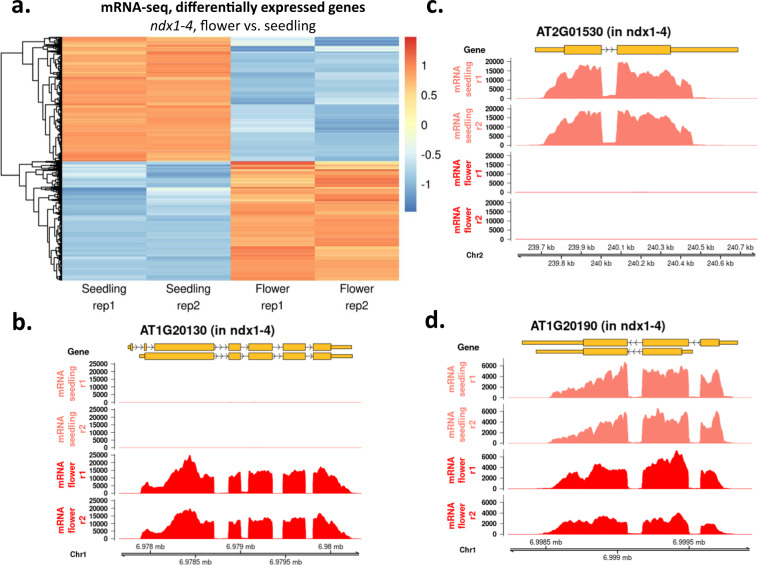
Differentially expressed genes in *ndx1-4* mutant seedlings and flowers. (**a**) Hierarchical clustering of log_2_ fold change values (seedling, *ndx1-4* vs. flower, *ndx1-4*) calculated by DESeq2 analysis. Orange shows upregulated genes, blue shows downregulated genes. Two independent biological replicates are shown. (**b**) Representative screenshots of downregulated genes in *ndx1-4* flowers. (**c**) Representative screenshots of upregulated genes in *ndx1-4* flowers. (**d**) Representative gene showing no differential expression in *ndx1-4* seedlings and flowers.

Expression changes of 31987 protein coding and noncoding genes and 34856 transposable elements were analysed. DEGs were visualized by hierarchical clustering in three different relations: (1) in inflorescence, we identified 269 (0.84%) up- and 145 (0.45%) downregulated genes in the *ndx1-4* mutant vs. Col-0 control samples (Fig. 3a). Representative genes are shown in Fig. 3b–d. (2) The transcriptomes of inflorescence and seedlings^6,16^ were compared in Col-0 (control) samples (Fig. 4a), identifying 5555 (17.37%) up- and 5618 (17.56%) downregulated genes in flowers relative to seedlings. Representative genes are shown in Fig. 4b–d. 3) The transcriptomes of inflorescence and seedlings^6,16^ were compared in *ndx1-4* mutant samples (Fig. 5a), identifying 5405 (16.9%) up- and 5645 (17.65%) downregulated genes in flowers vs. seedlings. In addition, 200 DEGs identified at the flowering stage between *ndx1-4* and Col-0 overlapped with DEGs identified in seedlings. Representative genes are shown in Fig. 5b–d. Collectively, the above analysis identified a great number of *ndx1-4*-specific and development-specific changes in gene expression profiles.

Differential expression of 34856 transposable elements were analysed in inflorescence samples. Only a small number of transposable elements were differentially expressed: we identified 33 (0.09%) upregulated and 12 (0.03%) downregulated transposable elements in the *ndx1-4* mutant vs. Col-0 control samples.

Comparing DEGs with TEs, 42 upregulated and 19 downregulated DEGs overlap with transposable elements, 7 upregulated and 3 downregulated DEGs overlap with differentially expressed transposable elements.

Description and accessibility of the generated datasets can be found in Tables 1, 2.

**Fig. 6:**
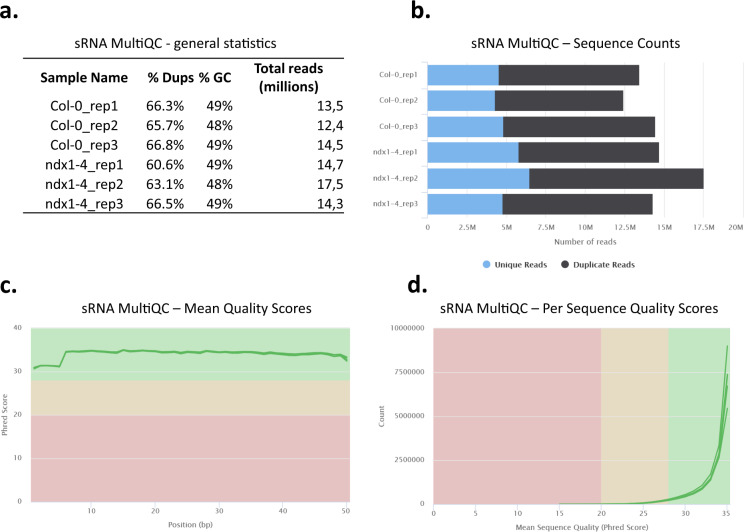
MultiQC analysis of sRNA-seq data from flowers. (**a**) General NGS statistics of sRNA-seq samples. (**b-d**) Same as in Fig. 2.

For the analysis of the noncoding transcriptome, sRNA libraries were prepared from 3-3 biological replicates according to Illumina’s NEBNext® Multiplex sRNA Library Prep protocol. For sRNA library preparations, equal amounts of total RNA extracts from 5 flowering tissues were pooled (to create one pool) for each biological replicate (see Tables 1, 2 for description of samples). Total RNA extraction was done as described above for mRNA-seq. The same total RNA prep was used for sRNA and mRNA sequencing. The sRNA-seq libraries were sequenced using an Illumina NextSeq500 instrument with 1 × 50 bp reads, generating 13.5–17.5 million reads per sample (Fig. 6a). Raw sequence data quality was assessed using FastQC and summarized reports were generated with MultiQC^9^. Duplication detection indicated 5–6 million unique reads per sample (Fig. 6b). Mean Phred quality scores for each read position in each sRNA sample were higher than 28 (indicative of ‘very good quality’) (Fig. 6c). Per sequence mean Phred quality scores were higher than 28 (indicative of ‘very good quality’) for at least 95% of reads in each sRNA sample (Fig. 6d).

**Fig. 7:**
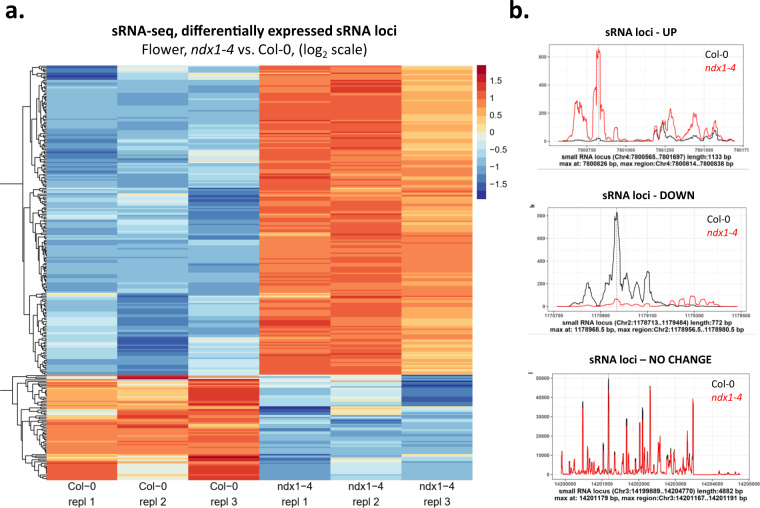
Differentially expressed sRNA loci in Col-0 and *ndx1-4* mutant *Arabidopsis* inflorescence. (**a**) Hierarchical clustering of log_2_ fold change values (flower, Col-0 vs. *ndx1-4*) calculated by DESeq2 analysis. Orange shows upregulated sRNA loci, blue shows downregulated sRNA loci. Three independent biological replicates are shown. (**b**) Representative screenshots of upregulated, downregulated sRNA loci in *ndx1-4* flowers. An sRNA locus with no transcriptional change is also shown (bottom panel).

The sRNA-seq data underwent additional processing using the sRNAnalyzer pipeline^17^. The Illumina adapters were initially removed by Cutadapt^18^, and the reads were then size-selected to range between 19–25 nt. These size-selected reads were subsequently aligned to a published comprehensive sRNA locus database^19^ (public data: www.ebi.ac.uk/ena/browser/view/PRJEB22276, additional material: https://github.com/seb-mueller/Arabidopsis_smallRNA_loci) using sRNAnalyzer. To determine differential expression of sRNA loci between Col-0 and ndx1-4 samples, DESeq2 was employed (p(adjusted) <0.05), and the hits were further filtered by absolute fold change (abs(FC) >1.5). For data visualization, reads were aligned to the *Arabidopsis thaliana* reference sequence (TAIR10) with bowtie2^20^. Finally, we utilized deepTools bamCoverage to generate RPKM (Reads per Kilobase per Million) normalized coverage files. Differential expression of 16517 sRNA loci were analysed. Our analysis identified 642 (3.89%) up- and 991 (6%) downregulated sRNA loci in the *ndx1-4* mutant relative to Col-0 control samples. At absolute fold change (abs(FC) >1.5) cutoff, we identified 375 (2.27%) up- and 179 (1.08%) downregulated sRNA loci that were visualized by hierarchical clustering (Fig. 7a). Representative sRNA loci are shown in Fig. 7b. Description and accessibility of the generated datasets can be found in Tables 1, 2.

## Data Records

The mRNA-seq and sRNA-seq datasets generated from *Arabidopsis* inflorescence tissues were deposited in Gene Expression Omnibus (GEO) under the accession number GSE223591^21^.

Genome browser (JBrowse) tracks containing relevant transcriptomic data are available at:

https://geneart.med.unideb.hu/pub/2023_ndx (login: ndx; password: athal23)

The mRNA and sRNA datasets generated from *Arabidopsis* seedlings can be accessed at GEO under the accession number GSE201841^16^ and in Supplementary Data 1–16 accompanying the related paper^6^.

Tables 1, 2 summarizes all transcriptomic experiments, NGS data, result tables, and identifiers to access relevant datasets. Table 3 lists all reference data used for analyses.

## Technical Validation

**Fig. 8:**
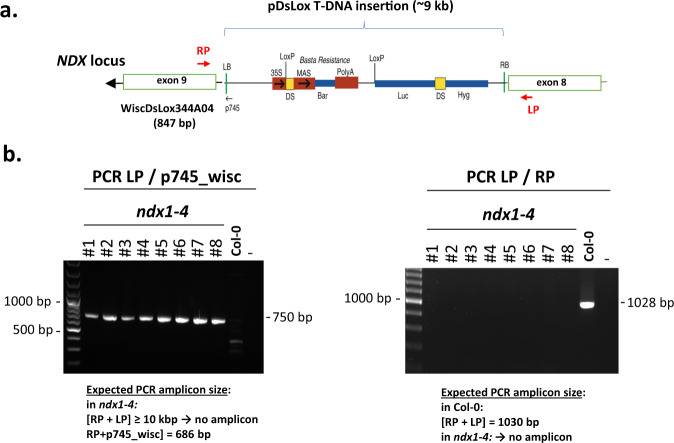
Genotyping of the *ndx1-4* mutant plant line (Col-0 background). (**a**) Schematic representation of T-DNA insertion into the NDX locus along with the genotyping PCR primers ndx1-4_gLP AAAGCTCGTGTTGGCTAAGTG; ndx1-4_gRP AGGTTTCTGCAAACACCAGTG; WISC-TDNA AACGTCCGCAATGTGTTATTAAGTTGTC. Expected sizes of PCR amplicons in Col-0 and *ndx1-4* mutant backgrounds are shown below. (**b**) Representative genotyping PCR in *ndx1-4* mutant and Col-0 plant lines. (−): no template control.

**Fig. 9:**
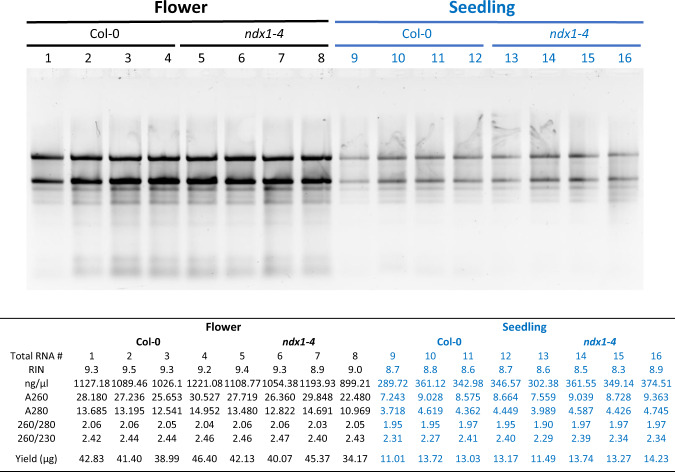
Total RNA isolated from Col-0 and *ndx1-4* mutant *Arabidopsis* seedlings and inflorescence samples. Upper panel: RNA integrity analysis performed by agarose gel electrophoresis. Dominant bands represent intact rRNAs. Lower panel: Purity and yield of total RNA samples measured by Nanodrop spectrophotometry and Agilent Bioanalyzer. OD 260/280 values > 1.8 and 260/230 values > 2.2 indicate the high purity of the samples. RIN: RNA integrity number.

**Fig. 10:**
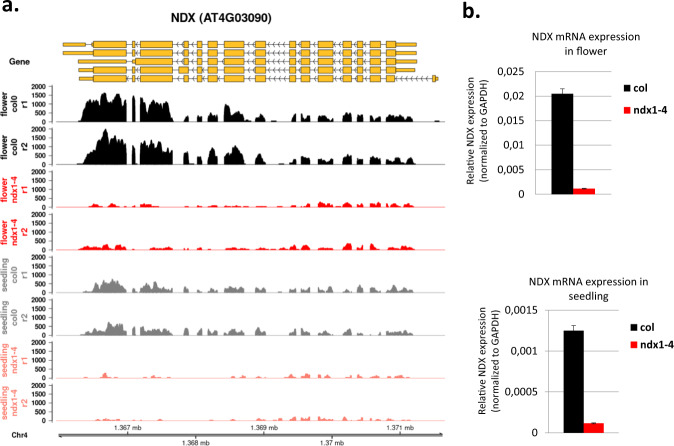
RT-qPCR validation of NDX mRNA expression in Col-0 and *ndx1-4* plants. (**a**) Genome browser screenshot showing mRNA-seq expression profiles over the NDX locus. Two independent biological replicates are shown. (**b**) RT-qPCR validation performed in Col-0 and *ndx1-4* flowers and seedlings. NDX mRNA expression levels were normalized to GAPDH1 expression, as described in the related paper^6^. NDX mRNA is barely detected in the *ndx1-4* mutant.

The genotypes of Col-0 and *ndx1-4* mutant *Arabidopsis* lines were confirmed by PCR using different combinations of genotyping primers designed for the NDX locus and the T-DNA (WiscDsLox344A04) insertion site (Fig. 8). The integrity, purity, and yield of extracted total RNA were determined by agarose gel electrophoresis and nanodrop spectrophotometry (Fig. 9) as well as Agilent bioanalyzer measurements (resulting in RIN values >9). Lack of mRNA expression from the mutagenized *ndx1-4* locus was confirmed by RT-qPCR analysis (Fig. 10). RNA samples were extracted from the 10-day-old seedling and inflorescence tissues (Col-0 and *ndx1-4* genotypes). To remove genomic contaminations, Dnase I treatments were performed based on manufacturer’s instructions (Ambion AM2222, www.thermofisher.com). These RNA samples were reverse transcribed with random hexamers using SuperScript IV reverse transcriptase (Thermo Fisher Scientific) as previously described^8^. For qPCR, we employed the ΔΔCt method and internal primers for normalization between samples. Primer sequences used for qPCR were as follows: NDX F AGCTGTAAAGTCAACTAACTGAGA; NDX R TCTAGATCCCATCTAACAAGAAACA; GAPDH1 F AGGAGCAAGGCAGTTAGTGGT; GAPDH1 R AGATGCGCCCATGTTCGTT. Real-time qPCR was subsequently conducted with a LightCycler 480 SYBR Green I Master mix (Roche) utilizing a QuantStudio 12 K Flex Real-Time PCR System (Thermo Fisher Scientific). NDX mRNA expression levels were normalized to GAPDH1 gene expression.

## Usage Notes

We have created a comprehensive database of coding and noncoding transcriptomes of *Arabidopsis* inflorescence and seedling tissues that can be used for downstream genomic analysis. The NGS tracks provided can be displayed directly in freely accessible genome browsers. The list of differentially expressed genes can be directly used for molecular pathway analysis, GO term analysis or gene set enrichment analysis. The identified differentially regulated sRNA loci can be integrated with histone modification, DNA methylation, and transcriptomic maps for in-depth epigenomic analysis. Altogether, the data source we generated in Col-0 and *ndx1-4* mutant plants is expected to lead us to a deeper understanding of the molecular function of NDX.

## Data Availability

No custom code was generated or applied for analysis of the genomic data presented. All software tools were referenced and used with default settings unless otherwise noted. Non-default parameters were as follows: FastQC (v0.11.9), multiqc (1.14): Quality check of mRNA-seq and sRNA-seq reads. Cutadapt (1.9.1): Adapter trimming of sRNA-seq reads (first pass parameters: -a AGATCGGAAGAGCACACGTCT -n 1 -e 0.2 -O 5 -m 1 --match-read-wildcards; second pass parameters: -g GTTCAGAGTTCTACAGTCCGACGATC -n 1 -e 0.125 -O 8 -m 1 --match-read-wildcards). After adapter trimming, reads were size-selected (using standard linux command line tools) to keep 18–25 nt reads. sRNAnalyzer pipeline: Alignment of sRNA reads (kit: NEB; min-length: 8; alignment type: multiple) to the A. thaliana sRNA locus database^19^. Salmon (1.7.0): Quantification of mRNA-seq reads has been performed with fragment GC bias correction (--gcBias). Duplicate reads were filtered out (default behaviour) for protein coding and noncoding genes. Duplicate reads were kept for transposable elements (--keepDuplicates). DESeq2 (1.24.0): Adjusted p-value < 0.05 for differential gene expression analysis, adjusted p-value < 0.05 for differential transposable element expression analysis, adjusted p-value < 0.05 for differential sRNA expression analysis. HISAT2 (2.1.0): mRNA-seq data alignment (reference: TAIR10) for visualization. bowtie2 (2.3.5): sRNA-seq data alignment (reference: TAIR10) for visualization. deepTools (3.3.0) bamCoverage: RPKM normalized coverage files of mRNA-seq (--binSize 1 --normalizeUsing RPKM) and sRNA-seq (-bs 15–smoothLength 45 --normalizeUsing RPKM) data for visualization in JBrowse.
